# Supplementary material for: The role of Seasonal Malaria Chemoprevention in the effect of Azithromycin on Child Mortality: A Secondary Analysis of the CHAT Cluster Randomized Clinical Trial
Source: medRxiv. 2025 May 2:2025.04.30.25326740. Preprint. [Version 1] doi: 10.1101/2025.04.30.25326740 (PMC12060949; doi:10.1101/2025.04.30.25326740)
Supplement: Supplement 1 [file NIHPP2025.04.30.25326740v1-supplement-1.pdf]

Supplementary Figures

Supplementary Figure 1- a) Mortality rate by treatment and SMC season and b) Mortality rate difference (AZ vs Placebo) by season adjusting for SMC coverage level

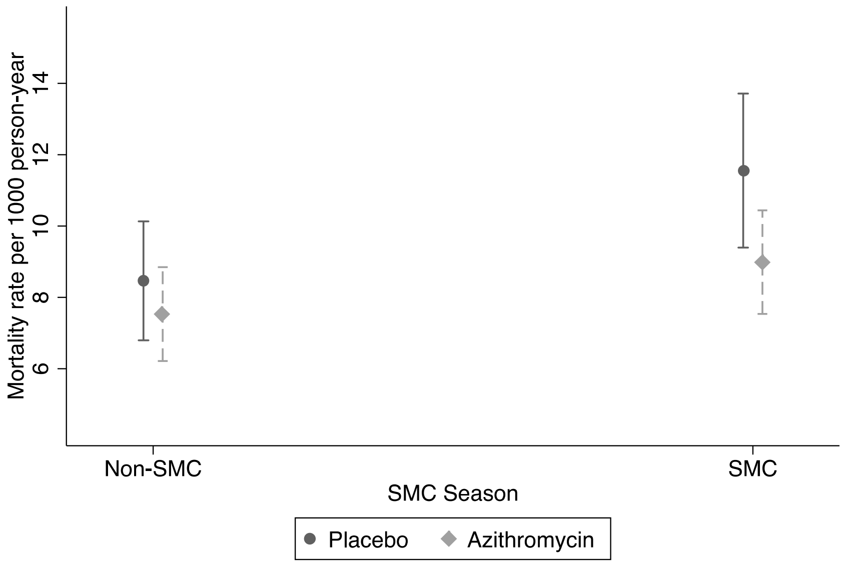

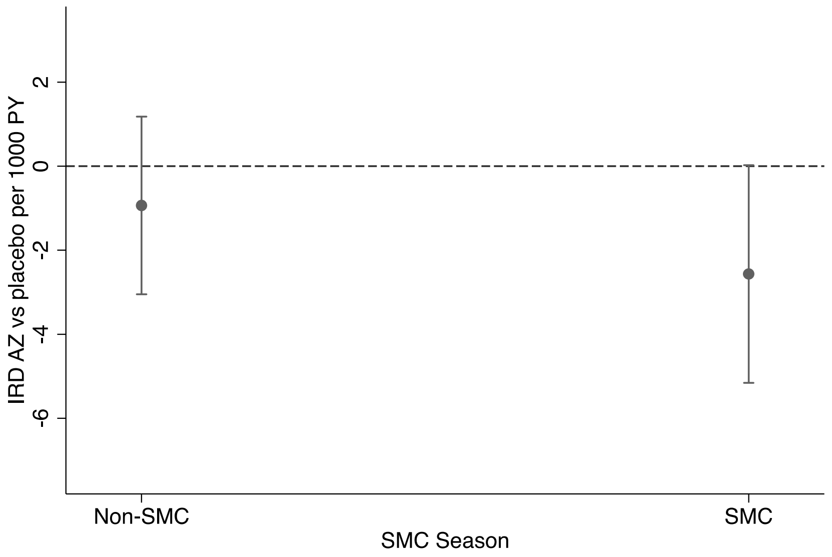

Supplementary Figure 2 - Mortality rate difference (AZ vs placebo) by quarter of year

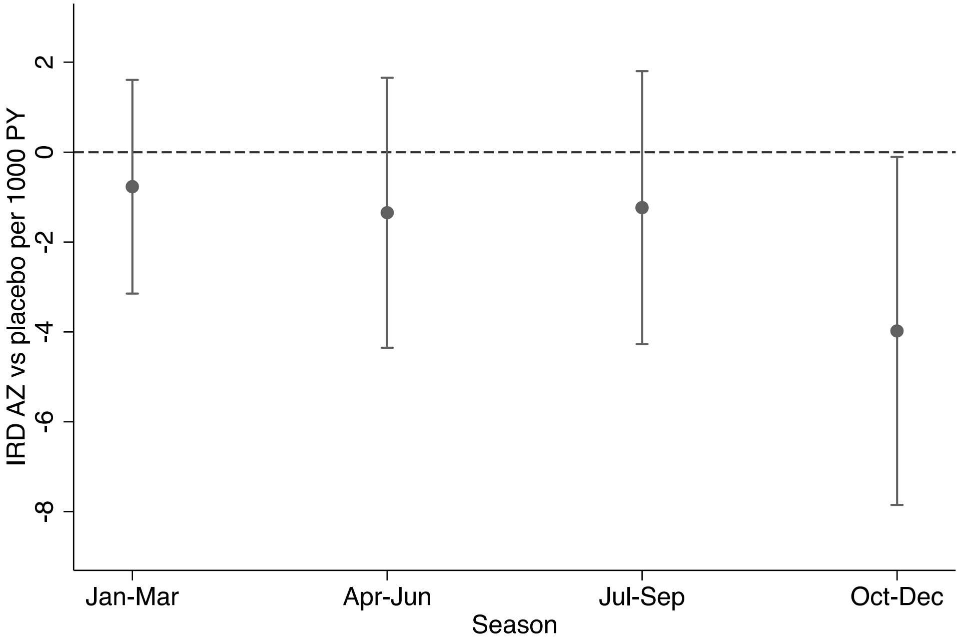

Note: P value for the interaction between AZ and quarter was 0.203 on the multiplicative scale and 0.148 on the additive scale

Supplementary Figure 3- Mortality Rate by Treatment and SMC Coverage: a) Continuous Scale and c) Threshold Level; Mortality Rate Difference (AZ vs. Placebo) by SMC Coverage: b) Continuous Scale and d) Threshold Level, Adjusting for Distance to Facility, Age of Children, and Their Interaction.

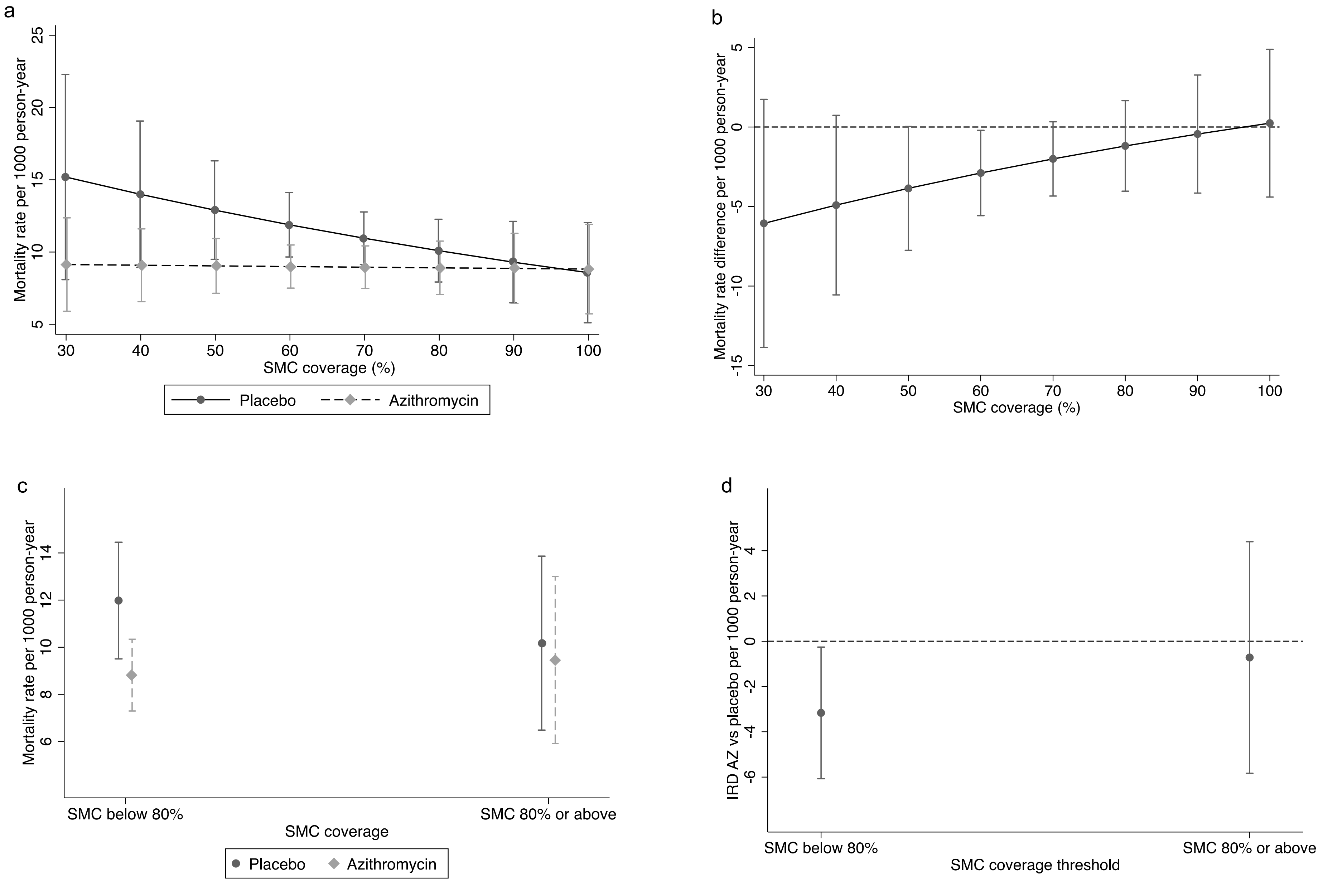

Supplementary Tables

Supplementary Table 1- Effect of Azithromycin vs. Placebo on Child Mortality by Season, Adjusting for SMC Coverage

	**Non-SMC (Jan-Jun)**	**SMC (Jul-Dec)**
Mortality rate per 1000 PY (all clusters)	7.9 (6.9 to 9.0)	10.3 (9 to 11.6)
Mortality rate per 1000 PY in AZ	7.5 (6.1 to 8.8)	9.0 (7.5 to 10.4)
Mortality rate per 1000 PY in Placebo	8.4 (6.8 to 10.1)	11.7 (9.5 to 13.9)
IRR (AZ vs placebo)	0.89 (0.68 to 1.15)	0.77 (0.6 to 0.98)
IRD (AZ vs placebo)	-0.97 (-3.07 to 1.14)	-2.7 (-5.3 to -0.07)
Number Needed to treat to avert one death	1037	371
Interaction Coeff multiplicative scale	0.87 (0.65 to 1.16), p= 0.335	
Interaction Coeff additive scale	-0.21 (-0.57 to 0.16), p=0.268	

Note: Table showing results from sensitivity analyses adjusting for coverage

Supplementary material

Supplement 1- CHAT Trial Manual of Operations
